# An Application of Real-Time PCR and CDC Protocol May Significantly Reduce the Incidence of *Streptococcus agalactiae* Infections among Neonates

**DOI:** 10.3390/pathogens11091064

**Published:** 2022-09-19

**Authors:** Tomasz Bogiel, Szymon Ziółkowski, Alicja Domian, Zuzanna Dobrzyńska

**Affiliations:** 1 Department of Microbiology, Ludwik Rydygier Collegium Medicum in Bydgoszcz, Nicolaus Copernicus University in Toruń, 85-094 Bydgoszcz, Poland; 2 Clinical Microbiology Laboratory, Dr. Antoni Jurasz University Hospital No. 1 in Bydgoszcz, 85-094 Bydgoszcz, Poland; 3 Department of Perinatology, Gynaecology and Gynaecologic Oncology, Chair of Pathomorphology and Placentology, Collegium Medicum, Nicolaus Copernicus University in Bydgoszcz, 85-094 Bydgoszcz, Poland; 4 Chair of Pathomorphology, Placentology and Clinical Hematopathology, Dr. Jan Biziel University Hospital No. 2 in Bydgoszcz, 85-168 Bydgoszcz, Poland

**Keywords:** *cfb* gene, congenital infections, GBS, neonatal infections, neonates, newborns, PCR, pregnancy, pregnant women, real-time PCR, *Streptococcus agalactiae*

## Abstract

*Streptococcus agalactiae* is an important human opportunistic pathogen, especially infectious for pregnant women and neonates. This pathogen belongs to beta hemolytic *Streptococcus* spp. representatives and accounts for a significant part of early infections in newborns, including serious life-threatening infections. This research investigated the usefulness of Centers for Disease Control and Prevention (CDC) protocol for *S. agalactiae* DNA detection in 250 samples of recto-vaginal swabs collected from pregnant women (at 35-37 weeks of gestation) and pre-cultured overnight in liquid medium. With an application of the CDC protocol-based real-time PCR, the *cfb* gene was detected in 68 (27.2%) samples compared to 41 (16.4%) for the standard culture-based methodology. The applied molecular method presented high sensitivity (100.0%) and specificity (87.1%). Therefore, it allowed for more precise detection of *S. agalactiae* bacteria, compared to the reference diagnostic method, culture on solid media with the following strain identification. The increased sensitivity of GBS detection may result in a reduced number of infections in newborns and leads to more targeted antimicrobial prophylaxis therapy of GBS infections in pregnant women. In addition, the use of the molecular method allows for a significant reduction in the time needed to obtain a result for GBS detection, and interpretation of the results is relatively simple. Therefore, it enables a faster intervention in case of a necessity of an antibiotic therapy introduction in pregnant women whose GBS status is unknown at the time of delivery.

## 1. Introduction

*Streptococcus agalactiae*, also known as group B *Streptococcus* (GBS) according to the Lancefield classification, belongs to Gram-positive cocci. These streptococci are typical opportunistic pathogens and commensals that constitute a part of microbiota of the oral cavity, nasopharynx, gastrointestinal tract and genitourinary system [1]. 

A GBS carriage is especially important in pregnant women. By an initial colonization of their reproductive tract, pathogens may ultimately reach the body of a newborn. The consequence of this may be a congenital infection of a neonate, causing, e.g., respiratory infections, meningitis, bacteremia and even death [2]. Factors that increase morbidity and mortality among newborns are a premature labor, a low birth weight and low levels of antibodies against the capsular polysaccharide of *S. agalactiae* [3]. Importantly, the risk of group B streptococcal infection may also occur in cases of caesarean section and also intact fetal membranes [4].

It should be remembered that GBS can also cause a serious threat to women giving birth. Meanwhile, the dynamics of GBS carriage among pregnant women is a very interesting issue [5]. Therefore, it is suggested to choose the most reliable method and possibly postpone a specimen sampling until at least 37 weeks gestation in the late pregnancy to increase the sensitivity of a GBS testing [6]. For example, a bacteria culture in enriched media and intrapartum polymerase chain reaction (PCR) methods present comparable sensitivities in GBS detection during late pregnancy when GBS screening is conducted no more than four weeks before labor. 

*Streptococcus agalactiae* identification is a challenge for modern microbiological diagnostics, especially taking into account the phenotypic and molecular diversity of the strains belonging to this species [7,8,9,10]. Moreover, in recent years, an increase in GBS resistance to selected drugs has been observed [11].

It has been calculated that a point-of-care intrapartum GBS PCR screening is associated with a significant reduction in an early-onset GBS disease incidence and antibiotic use [12,13]. In a group of newborns it definitely balances the additional PCR-based diagnostic costs by the reduction in early-onset GBS disease treatment expenses [14]. Therefore, European countries recommend intrapartum antimicrobial prophylaxis application based on the results of intrapartum GBS screening strategy using rapid real time testing [15]. However, it is underlined that this testing has to be applied in reasonable advance to labor and with a strict regime to allow for adequate exposure to antimicrobials and their sufficient activity for protection of newborns [16]. 

One of the recommendations of the Centers for Disease Control and Prevention (CDC) from 2010 is that vaginal and/or rectal swabs of women between 35 and 37 weeks of gestation should be pre-incubated overnight in Todd–Hewitt (or corresponding) broth, then plated on solid media and suspected colonies should be identified to the species level [17]. However, it is believed that this method is less sensitive and definitely has a longer time to result (TTR) compared to the molecular biology test [18]. Moreover, access to this methodology, and to any GBS detection method and scheme applied worldwide, is very diverse [19].

Meanwhile, the constant development of diagnostic methods makes it possible to detect GBS more accurately and quickly. A test characterized by higher sensitivity and shorter TTR is the real-time PCR, based on the amplification of species-specific and conserved genes (e.g., the *cfb* gene) [20]. Owing to this methodology, it is possible to also detect non-hemolytic and non-pigmented GBS strains which, according to some reports, occur in 1–4% of pregnant women [21]. Moreover, it has been previously confirmed that real-time identification of GBS with an application of PCR reduces the intrapartum use of antibiotics, especially in case of women with negative GBS status or a prolonged rupture of the membranes. Taking into account a time necessary for PCR investigation and average delivery time, it leads to a reduction in antibiotic use during labor [22]. Meanwhile, reduction in the use of antimicrobial drugs following the rapid detection of *S. agalactiae* carriage by real-time PCR assay at delivery is also of great importance [23]. It reduces not only a number of inadequate antimicrobial treatments aimed to prevent the early onset of GBS disease but also costs of treating potentially GBS-infected neonates [19]. 

A real-time PCR-based assay is highly accurate to identify intrapartum GBS carriers at point of care. This method can enhance the exact identification of candidates for intrapartum antibiotic prophylaxis [24,25], including women with pre-term rupture of membranes or pre-term labor [26,27] or not investigated during pregnancy [28].

Therefore, the aim of the study was to assess the usefulness of the real-time PCR method for the detection of *S. agalactiae* DNA in vaginal/rectal swabs of pregnant women. A comparison of the results obtained with the standard GBS identification method may be helpful for a selection of faster, more sensitive and specific method, which still allows for a reliable identification of this species.

## 2. Results

**Figure 1:**
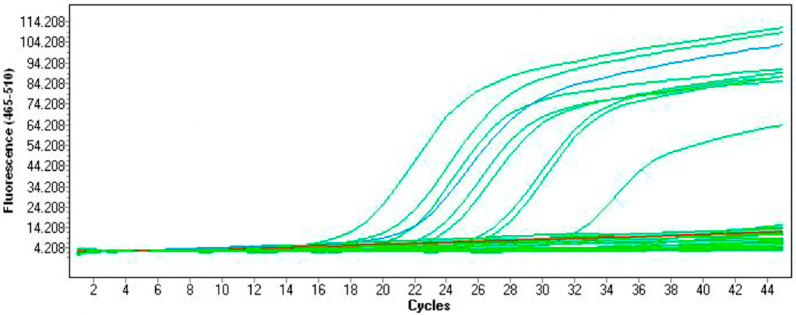
Plot showing the curves obtained during real-time PCR amplification: blue curve—positive control; red line—negative control (molecular biology grade water); green lines—the investigated strains, either positive showing fluorescence or negative in the fluorescence background.

The studies carried out allowed for the determination of the GBS carriage incidence among pregnant women in the population of a certain area in Poland. The positive result of real-time PCR was obtained for 68 (27.2%) samples, while for the culture method the corresponding values were 41 (16.4%). An example of the plot showing curves obtained during real-time PCR amplification is shown in Figure 1.

Table 1 compares the real-time PCR results with the results of culture on solid media, which is one of the stages of a routine diagnostic procedure dedicated for GBS investigation.

The correlation between the results obtained with both methods amongst the samples collected from pregnant women is also shown in Table 1. 

The presence of *S. agalactiae* DNA was additionally found in 27 (10.8%) samples for which no growth of GBS was observed on the solid medium. The real-time PCR was performed again for these samples together with 39 other arbitrarily chosen (27 negative and 12 positive) samples. The results of this repeated investigation were concordant with those in the first reaction. Therefore, they were considered true positive or true negative, respectively.

The statistical analysis showed that the sensitivity of the real-time PCR, compared to a reference bacterial culture methodology was 100.0%, while the specificity was 87.1%. The positive predictive value (PPV) was 60.3%, while the negative predictive value (NPV) was 100.0%. 

## 3. Discussion

*Streptococcus agalactiae* can cause infections in humans at different ages, with pregnant women and newborns being the most vulnerable. They may develop an early-onset or late-onset disease (EOD/LOD), including a neonate death. The most common reason for this is an increasing recto-vaginal colonization of pregnant woman, which is associated with a possibility of a vertical transmission of GBS during a pregnancy or a delivery. Despite the use of intrapartum prophylactic antibiotic therapy, GBS infections in newborns are still a significant problem [29]. It is related to the pre-term labor, unknown GBS status of some pregnant women, and also their increasing incidence of resistance to antibiotics, especially to those from the non-beta lactams group [30]. Therefore, screening tests of pregnant women between 35–37 weeks are one of the most important aspects in the prevention of GBS infections. Increasingly, in addition to the recommended culture-based methods (on blood agar preferably), molecular biology methods are used. They usually involve real-time PCR, either “in house” assays, dedicated to a number of PCR devices or molecular platforms. All of them differ in sensitivity and specificity of the results [1,31,32,33,34,35,36,37,38,39,40,41,42,43].

In our study, the presence of the *cfb* gene was found in 68 (27.2%) samples, which is associated with 100.0% sensitivity for GBS detection and a specificity of 87.1%. Using the culture method, the presence of *S. agalactiae* was found in 41 (16.4%) samples only. Bakhtiari et al. [44] also found GBS DNA more frequently than the living bacteria in culture of vaginal and rectal swabs collected from 375 pregnant women, at 28–38 weeks of gestation (11.2% vs. 9.3%).

High sensitivity of the real-time PCR-based tests in the identification of *S. agalactiae* was also confirmed in the Escobar et al. [45] and Gerolyma et al. [46] studies. Their first studies showed that detection of low concentrations of genomic DNA using quantitative PCR (qPCR) is also possible with high sensitivity (95.4%). Therefore, this may also allow for the determination of GBS DNA directly from vaginal swabs, without the necessity of their pre-culture. PCR performed at the time of a labor also had a higher detection rate (although non-significant) and a PPV value in identification of a high load of vaginal GBS DNA compared with recto-vaginal culture of swabs derived from laboring women [47]. 

The reliable investigation of low GBS DNA concentrations was confirmed in research conducted by Ellem et al. [48]. Using the qPCR method for the analysis of the swabs directly, sensitivity of the results was 98.4%. The sensitivity reached 100% for the results obtained for samples previously multiplied in broth. Of note, the CDC recommendations does not recommend GBS detection directly from a vaginal/rectal swab without a pre-culture step [17].

In their research using culture and PCR, Ge et al. [49] compared the GBS colonization percentage for the most numerous collection of 16,184 pregnant women from different age groups. Although they did not determine the sensitivity and specificity of this PCR technique, they found this molecular method to be much more sensitive and specific. Using the PCR method, this was evidenced by more than doubled prevalence of GBS colonization in the groups of pregnant women examined.

Similarly, Zietek et al. [50] assessed the usefulness of the real-time PCR in relation to the culture-based methods. The sensitivity and specificity of PCR assay calculated by them was 81.8% and 86.9%, respectively. These values are lower than those obtained in our research, but still indicate the advantage of this molecular method over culture. 

An application of the culture method only leads to missed false negative carrier individuals. Therefore, it is recommended to combine PCR assay and the conventional culture method in order to increase the reliability of *S. agalactiae* detection results. In turn, some authors discuss the issue of false negative PCR results and its limited usefulness for a routine GBS testing [51]. 

As shown previously, the choice of a gene for a particular method of GBS detection is of great importance in this case [20,36]. In the study conducted by Carrillo-Ávila et al. [18], 320 recto-vaginal swabs were collected and the assessment involved investigation of two genes: the *cfb* gene, as in the CDC recommendations, and also the *sip* gene. The sensitivity and specificity results of the applied qPCR technique, compared with culture as the gold standard, were 93.6% and 94.6%, respectively. False negative results using the real-time PCR method were probably due to low bacterial numbers, which correspond to low DNA concentration in the samples. Another reason for this could be the modified DNA extraction procedure, carried out contrary to the CDC recommendations.

Goudarzi et al. [52] collected recto-vaginal swabs from 200 patients. Although they achieved high specificity (96.1%), the sensitivity (72.7%) was quite low compared to the results of other authors’ research. They extracted the genetic material from the inoculum obtained by placing the collected swabs in phosphate-buffered saline (PBS). This could have had a significant impact on the appearance of false negative results and the observed decreased sensitivity. Meanwhile, Guo et al. [53] obtained only one false negative result after performing the PCR reaction. In their study, the density of the bacterial suspension was increased to four in the McFarland scale. Despite this model, the sample was still negative. The authors suspected that the reason for this phenomenon might be the presence of deletion mutants concerning the *cfb* gene, which enables amplification of the target DNA due to lack of compatibility of primers/probes with a native GBS DNA lack of hybridization and inability to initiate the amplification of the exact DNA sequence. Another reason for this is the presence of the new and modified CAMP factor II. Detailed research concerning these deletion mutants was carried out by Tickler et al. [54]. They tested 145 samples for the presence of GBS. Their strains were analyzed with pulsed-field gel electrophoresis (PFGE) and whole-genome sequencing. It was proved that various size deletions were located within the DNA binding site for the primer and probe. It led to a lack of amplification, causing false negative results. Owing to the limited research on the subject of deletion mutants concerning the *cfb* gene, it is recommended that these strains should be subjected to further studies [55].

In the present study with use of the real-time PCR method, positive results were obtained for 27 (10.8%) samples that gave negative results in culture on a solid medium. Despite the fact that the culture on solid media is believed to be the reference method, it is fraught with numerous disadvantages. One of them is the difficulty in identifying non-hemolytic and/or non-pigmenting GBS strains. Moreover, due to abundance of other growing bacteria, beta hemolysis of the blood-supplemented medium may not be noticeable, which can additionally lead to false negative results [21]. Other reasons for a limited culture method sensitivity might be the low number of streptococcal cells in the swab, due to an improper sample collection, a reduced viability of the GBS and inappropriate sampling or material transporting [39]. Surprisingly, the differences in a prevalence of a GBS carriage may also result from the manner of swab collection. The results of the study conducted by Bidgani et al. revealed that the frequency of GBS culture-positive rectal samples was higher than vaginal, while for the PCR the corresponding results were exactly opposite [56]. However, the samples included in our study were collected according to recommendation, from both localizations using one swab.

Our re-analysis of the samples with discordant results between the methods applied, allowed us to consider the PCR positive results true positive. A similar situation was noted in Goudarzi et al. [52], in which positive real-time PCR results were obtained for seven samples, while no growth was observed on solid media. Despite being considered statistically a false positive, researchers confirmed the presence of *S. agalactiae* in the samples. According to them, the patients from whom the swabs were derived could have taken antibiotics or had an overgrowth of gut microbiota. This could affect the false negative results of a bacterial culture. 

On the other hand, Bergseng et al. [57], who conducted research on 251 samples, found a positive result of real-time PCR and negative culture in the samples derived from two pregnant women. However, they were investigating a different fragment of the target gene sequence—*sip*, not the *cfb* gene, as in our research. To confirm this result, they additionally performed the electrophoresis of real-time PCR products, together with the classic PCR reaction, aiming at assessing the occurrence of the additional *cylE* gene. Ultimately, the results allowed for identification of the GBS carrier in these two women on the basis of positive real-time PCR results.

Particular attention should be given to studies comparing the effectiveness of GBS identification among different manufacturers of analytical instruments and tests used on analytic platforms. One of these studies was performed by Relich et al. [58], comparing the BD MAX GBS (Becton Dickinson, Franklin Lakes, NJ, US), ARIES^®^ GBS (Luminex Corporation, Austin, TX, US), Illumigene^®^ Group B *Streptococcus* (Meridian Bioscience, Cincinnati, OH, US) and Xpert^®^ GBS LB (Cepheid Inc., Vira Solelh, Maurens-Scopont, France) tests. The research was carried out on 299 samples and the results then subjected to multidirectional analysis. For this purpose, the sensitivity, specificity, PPV, NPV, method efficiency and reaction time, were compared. A study by Relich et al. [58] showed that all the investigated assays had a very similar sensitivity, around 97.0%. From the compared tests, the Xpert^®^ GBS LB assay had the shortest (1 h and 12 min) while the BD MAX GBS instrument had the highest (2 h and 10 min) analysis time.

Another study, evaluating GBS identification tests in pregnant women, was that carried out by Miller et al. [59] that compared the AmpliVue™ GBS (Quidel Corporation, San Diego, CA, US), BD MAX GBS and Illumigene^®^ Group B *Streptococcus* tests. The researchers analyzed a total of 200 vaginal or rectal swabs for the presence of GBS DNA, and then the results were carefully analyzed. The sensitivity of the methods was: 96.4% for AmpliVue™ GBS, 100% for BD MAX GBS and 90.9% for Illumigene^®^. The researchers also focused on the differences in outcomes among the nucleic acids amplification tests that were used. Discrepancies were found for 22 (11.0%) samples. To verify the results, they re-tested the samples and 10 of the 22 analyzed samples were positive in two of the three methods. The reason for the differences in their results was probably the method detection limit, which amounted to 1.39 × 10^6^ CFU/mL for AmpliVue™ GBS, 2 × 10^4^ CFU/mL for BD MAX GBS and 2.56 × 10^4^ CFU/mL for Illumigene^®^.

Analysis of the results presented in the studies performed by Relich et al. [58] and Miller et al. [59], together with our own previous research, indicates that certain compliance should be noted. The sensitivity of the methodologies was very high for each case and the highest (98–100%) was achieved with the BD MAX GBS test. This test has also been shown to have the lowest detection limit. However, BD MAX GBS testing requires the longest TTR of all the tests that were compared. Moreover, it allowed for the analysis of up to 24 samples in one reaction. Comparing the BD MAX GBS test with the GBS identification used in this study, it should be stated that the test based on the CDC methodology also shows 100% sensitivity. In addition, CDC-recommended methodology is faster and, depending on the available devices, allows for simultaneous analysis of up to 382 samples in one run.

A DNA isolation procedure is also of great importance for the sensitivity of GBS detection results. The PathogenFree DNA Isolation Kit (GeneProof, Brno, Czech Republic) used in our study is certified for in vitro diagnostics and the whole protocol requires approximately one hour. Adding the time necessary for reagent preparation and the real-time PCR protocol based on the CDC recommendations, the entire procedure takes altogether less than 3 h. For comparison, the incubation steps plus the time necessary for bacteria identification when using solid medium culture is mostly between 48 and 72 h [60]. In addition, owing to the use of the real-time PCR method, it is possible to analyze many samples simultaneously. 

Berry et al. [61] compared the efficiency of GBS identification tests based on the BD MAX and Panther Fusion (Hologic Inc., Marlborough, MA, US) platforms. Both tests detect GBS based on the *cfb* gene and real-time PCR method. The capacity of these devices during one reaction of the analysis was 24 and 84 samples, respectively. This shows that the identification of GBS based on the CDC protocol using the LightCycler^®^ 480 II (Roche) is a much more efficient method than that based on the BD MAX system and comparable to that based on the Panther Fusion platform.

In the present study, the threshold cycle (Ct) values were in the range of 13.98–42.89. High Ct values (above 30) were mainly observed in the samples that showed no presence of GBS in solid medium culture. Therefore, it proves a low concentration of *S. agalactiae* DNA in the tested samples. The reason for this is probably a low number of bacterial cells collected with a swab, also causing the false negative result of bacteria culture. This phenomenon is also confirmed by the observations of other authors, who obtained high PCR Ct values for the samples negative in the culture [59,61].

In the case of the qPCR method, it is important to use properly the specific primers that enable amplification of the appropriate gene fragment. The issue may be point mutations or deletions within the gene sequences studied, along with non-specific interactions with DNA of a similar sequence. Therefore, it is important to check the primers for the specificity of the reaction. Such an analysis was performed by Carrillo-Ávila et al. [18], who designed their own primers for the analysis of the *S. agalactiae cfb* gene. Then, they were checked for the specificity of the amplification with *Streptococcus pyogenes* (AF079502), *Streptococcus uberis* (U34322), *Streptococcus canis* (AF488802) and *Streptococcus faecalis* (29374661) DNA.

It should also be remembered that the presence of inhibitors in the clinical specimen (e.g., bile salts, polysaccharides, blood, serum, plasma, IgG, hemoglobin, lactoferrin) can lead to false results when using the qPCR method. Moreover, various errors of the analytical phase may occur, such as an addition of inappropriate volumes of the reaction mixture or its improper mixing. A proper sealing of the reaction plate with an optical foil is also important for GBS identification by qPCR. Inappropriate sealing may cause the evaporation of the reaction mixture, and as a consequence, false results of the investigation. Other analytical mistakes are distortion of the thermocycler temperature or problems with a light source or filters for the fluorescence emission in the tested sample.

Summarizing, the results of the study prove the usefulness of molecular biology techniques in the evaluation of *S. agalactiae* carriage, including the real-time PCR method. The *cfb* gene used to identify GBS resulted in being 100% specific. Comparing the results of our own research with data from the literature indicates that the sensitivity of the qPCR technique was also very high. It proves the effectiveness of the methodology applied in this study, as previously noted also by other authors.

## 4. Materials and Methods

### 4.1. Origin of Samples and Their Selection Criteria

The study involved 250 samples of vaginal/rectal swabs incubated overnight in Todd–Hewitt medium. All the swabs were obtained from women at 35–37 weeks of pregnancy and collected in a routine prenatal GBS screening procedure. The real-time PCR was conducted using the same swab as for the culture method (exactly the same vagino-rectal swab was used for comparison of the methods, as the main study purpose).

The DNA isolated from the reference *S. agalactiae* strain, derived from the German Collection of Microorganisms and Cell Cultures (DSMZ No. 2134), served as positive control for the *cfb* gene detection.

### 4.2. Bacterial Culture and Strain Identification

After the collection step, the samples were pre-incubated in Todd–Hewitt broth at 37 °C for 18–24 h. Then, they were plated on either blood-supplemented agar or Granada culture media (*bio*Mérieux) for the assessment of GBS presence. After an overnight incubation, typical colonies of beta hemolytic or pigmenting bacteria were identified with the application of a MALDI-TOF MS device (Bruker) routinely applied in the Microbiology Department University Hospital No. 1 in Bydgoszcz, Poland, or corresponding methodology. 

### 4.3. DNA Isolation

DNA was isolated from the samples and from the reference *S. agalactiae* strain with application of the PathogenFree DNA Isolation Kit (GeneProof, Brno, Czech Republic) following the manufacturer’s instructions. To confirm the DNA isolation accuracy, the DNA samples were checked spectrophotometrically (Photometer, Eppendorf, Germany). The DNA samples were stored at −20 °C before their further use in real-time PCR.

### 4.4. Detection of the cfb Gene

The presence of the *cfb* gene was determined by the methodology recommended by CDC, using real-time PCR method in the LightCycler 480 II Instrument (Roche Diagnostics, Basel, Switzerland). Positive and negative (molecular biology grade sterile water) controls were used simultaneously for each run. The DNA isolated from the *S. agalactiae* DSMZ 2134 strain served as PCR positive control. Reactions were performed with the application of molecular-biology-grade sterile water (EURx, Gdansk, Poland), the *cfb* F and the *cfb* R primers (Genomed, DNA Sequencing and Oligonucleotide Synthesis Laboratory, Institute of Biochemistry and Biophysics of the Polish Academy of Sciences, Warsaw, Poland), and FAM-labeled hydrolysis probe, all described in Table 2, and the 2× PerfeCTa^®^ qPCR ToughMix^®^ reaction mixture (A&A Biotechnology). The reaction volume of one sample was 25 µL with 12.5 µL of ToughMix^®^ added to reach the final concentration of 1×; primers and probe were used at the final concentration of 200 nM (0.5 µL of each primer at the initial concentration of 10 µM and 5 µL of the probe at the initial concentration of 1 µM). The remaining volume was water (4 µL) and DNA template (2.5 µL). The amplification program consisted of initial activation at 95 °C for 10 min, followed by 45 cycles of amplification, each consisting of 15 sec at 95 °C and 60 sec at 60 °C. 

For all the samples that gave discordant results between culture and real-time PCR, the latter was repeated for verification (data not shown). The final PCR result was considered positive if there was a typical amplification curve in at least one of the investigations.

Moreover, for the 39 arbitrarily selected samples, PCR duplicates were performed to confirm the repeatability of the results, giving the consent results in each case (data not shown).

### 4.5. Statistical Methods

The sensitivity and specificity of CDC recommendations/real-time PCR methodology in relation to solid medium culture (the “gold standard” for GBS identification) were calculated in the XLSTAT program (Addinsoft) using standard calculation formulas with a 95% confidence interval. The positive and negative predictive values were also calculated according to commonly available formulas.

## 5. Conclusions

The research shows that the methodology based on CDC recommendations and real-time PCR protocol for the identification of the *cfb* gene can be successfully used to identify group B beta hemolytic streptococci in the samples of recto-vaginal swabs, derived from pregnant women, and pre-cultured overnight in a liquid medium. Very high sensitivity of the results, which reaches 100%, allows for the detection of GBS with much greater accuracy than the application of a bacteria culture. Moreover, the sample preparation and investigation is quite easy, while the interpretation of results is relatively simple. Thus, using this methodology, the TTR can be significantly shortened. This method also allows for simultaneous analysis of many samples. Knowing that the application of this molecular biology-based method can increase sensitivity of GBS testing results, it should be implemented in microbiology laboratories worldwide. It may reduce the number of congenital infections and lead to a more targeted antibiotic prophylaxis therapy of pregnant women, especially those without known GBS status at the time of delivery.

## Figures and Tables

**Table 1:** Comparison of *S. agalactiae* detection results obtained with an application of the real-time PCR methodology and the culture on solid media (*n* = 250).

		Real-Time PCR (*cfb* Gene)		
**Culture**	**Result**	**Positive**	**Negative**	**Total**
	**Positive**	41 (16.4%)	0 (0.0%)	41 (16.4%)
	**Negative**	27 (10.8%)	182 (72.8%)	209 (83.6%)
	**Total**	68 (27.2%)	182 (72.8%)	250 (100.0%)

**Table 2:** Specification of the primers and probe applied in real-time PCR.

PCR Primer Name	Primer Sequence 5′→ 3′	Concentration (nM)
*cfb*-F	GGGAACAGATTATGAAAAACCG	200
*cfb*-R	AAGGCTTCTACACGACTACCAA	
**PCR Probe Name**	**Probe Sequence 5′→3′**	
*cfb*-probe	5′-FAM-AGACTTCATTGCGTGCC AACCCTGAGAC-3′-BHQ1	

## Data Availability

The data presented in this study are available on request from the corresponding author.
